# AI decision support for increasing prostate biopsy efficiency: a retrospective multicentre, multiscanner study

**DOI:** 10.1007/s00330-026-12361-6

**Published:** 2026-02-20

**Authors:** Nikita Sushentsev, Zobair Arya, Jobie Budd, Amy Frary, Nadia Moreira da Silva, Mirjana Ferrer Rodriguez, Paul Burn, Richard Hindley, Nikhil Vasdev, Mohamed Ibrahim, Alison Bradley, Adrian Andreou, Sidath Liyanage, Raj Persad, Jonathan Aning, Alexander B. C. D. Ng, Aqua Asif, Veeru Kasivisvanathan, Tristan Barrett, Mark Hinton, Anwar Roshanali Padhani, Aarti Shah, Lucy Davies, Antony Rix, Evis Sala

**Affiliations:** 1 Department of Radiology, Addenbrooke’s Hospital and University of Cambridge, Cambridge, UK; 2 Lucida Medical Ltd, Cambridge, UK; 3 Somerset NHS Foundation Trust, Taunton, UK; 4 University of Winchester, Winchester, UK; 5 Hampshire Hospitals NHS Foundation Trust, Winchester, UK; 6 Department of Urology, Hertfordshire and Bedfordshire Urological Cancer Centre, Lister Hospital, Stevenage, UK; 7 School of Life and Medical Sciences, University of Hertfordshire, Hatfield, UK; 8 East and North Hertfordshire Teaching NHS Trust, Stevenage, UK; 9 Royal Cornwall Hospitals NHS Trust, Truro, UK; 10 Royal United Hospitals Bath NHS Foundation Trust, Bath, UK; 11 Mid and South Essex NHS Foundation Trust, Southend, UK; 12 North Bristol NHS Trust, Bristol, UK; 13 Division of Surgery and Interventional Science, UCL, London, UK; 14 Centre for Urology Imaging, Prostate, AI and Surgical Studies (COMPASS) Research Group, Division of Surgery and Interventional Science, UCL, London, UK; 15 Department of Urology, University College London Hospitals NHS Foundation Trust, London, UK; 16 Paul Strickland Scanner Centre, Mount Vernon Hospital, Northwood, UK; 17 Dipartimento Diagnostica per Immagini e Radioterapia Oncologica, Policlinico Universitario A. Gemelli IRCCS, Rome, Italy; 18 Dipartimento di Scienze Radiologiche ed Ematologiche, Università Cattolica del Sacro Cuore, Rome, Italy

**Keywords:** Prostate cancer, Magnetic resonance imaging, AI (artificial intelligence)

## Abstract

**Objectives:**

To develop and retrospectively validate an artificial intelligence-based decision support system (AI-DSS) for optimising prostate biopsy decisions and improving benefit-to-harm ratios.

**Materials and methods:**

This retrospective, multicentre, multiscanner study used data from 1022 patients. An AI-DSS integrating PI-RADS scores, automated prostate-specific antigen density (PSAd), and deep-learning imaging risk scores was developed on 770 cases and validated on an independent cohort of 252 men from six UK centres. The AI-DSS performance was benchmarked against the real-world clinical decisions (reference standard) using grade selectivity, biopsy efficiency, and selective biopsy avoidance as outcome measures. Biopsy-proven detection of grade group (GG) ≥ 2 disease was the reference standard.

**Results:**

In the validation cohort of 252 patients (mean age, 67.3 years), 137 underwent biopsy and 79 (31%) harboured ≥ GG2 disease. Compared to the reference standard, the AI-DSS at the 31% cancer detection rate (CDR) would have avoided 28 biopsies while missing one ≥ GG2 cancer. This corresponded to a 70% increase in grade selectivity (from 4.6 to 7.8), 79% increase in biopsy efficiency (from 1.4 to 2.5), and a 143% increase in selective biopsy avoidance (from 2.8 to 6.8). At the reduced CDR of 30%, grade selectivity, biopsy efficiency, and selective biopsy avoidance increased by 172%, 236%, and 475%, with four ≥ GG2 cancers missed.

**Conclusion:**

An AI-DSS that integrates clinical and advanced imaging data improves the benefit-to-harm ratio of prostate biopsy decisions in a retrospective setting. Future prospective validation as part of real-world clinical workflow is required to enable clinical implementation.

**Key Points:**

***Question*** *Current prostate cancer diagnostic pathways result in fewer unnecessary biopsies. Can an AI decision support system (AI-DSS) further improve biopsy efficiency for detecting significant cancer?*

***Findings*** *An AI-DSS avoided 28 biopsies in a 252-patient cohort, increasing grade selectivity, biopsy efficiency, and selective biopsy avoidance by 70%, 79%, and 143%, respectively.*

***Clinical relevance*** *Integrating an AI-DSS into clinical workflows may further reduce unnecessary prostate biopsies and overdiagnosis of indolent disease, thus potentially improving the efficiency of the prostate cancer diagnostic pathway.*

**Graphical Abstract:**

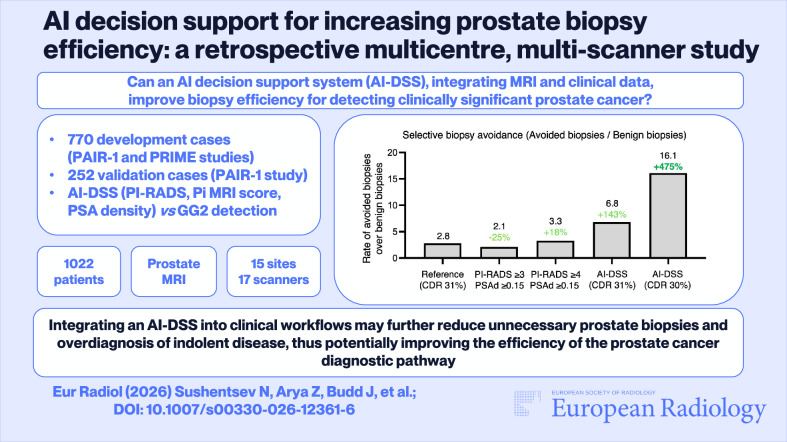

## Introduction

Pre-biopsy multiparametric magnetic resonance imaging (mpMRI) is the recommended first-line diagnostic tool in patients with suspected localised prostate cancer (PCa) [1–3]. Accordingly, the use of pre-biopsy MRI has been growing steadily [4, 5], a trend expected to continue given the increasing PCa incidence [6] and the potential introduction of MRI-driven population screening [7]. This growth puts considerable strain on the diagnostic workforce [8], indicating the need for optimisation of the MRI-based PCa diagnostic pathway [9].

Key limitations of the current pathway include high inter-reader variability [10], which limits equitable access to high-quality MRI interpretation outside expert centres. Another limitation is the low positive predictive value of MRI for detecting grade group (GG) ≥ 2 disease [10, 11]. There is a critical need to reduce false-positive cases to minimise unnecessary biopsies and thereby mitigate the overdiagnosis and overtreatment of indolent disease [12].

AI-based decision support systems (AI-DSS), trained on diverse, expert-annotated datasets, are promising tools for addressing these challenges. Although AI tools provide at least non-inferior performance compared to expert radiologists [13, 14], further research is needed to assess their potential to improve the benefit-to-harm ratios for men undergoing prostate biopsy [12]. Biopsies detecting ≥ GG2 disease are considered a benefit of the pathway, whereas detecting either GG1 PCa or no cancer at all (G0) is considered unproductive and potentially harmful [12].

In this study, we first developed an AI-DSS that calculates prostate volume, prostate-specific antigen density (PSAD), and imaging-based PCa risk scores to predict whether a biopsy would result in the detection of ≥ GG2 disease. We then retrospectively applied this system to an independent pre-biopsy cohort from six UK centres to assess its potential to improve biopsy efficiency, grade selectivity, and the selectivity of biopsy avoidance.

## Materials and methods

### Dataset characteristics

This retrospective, exploratory study used clinical and mpMRI data from 1022 patients investigated for suspected clinically localised PCa. Of those, 770/1022 (75%) cases were used for AI-DSS development, with the remaining 252/1022 (25%) cases used for validation after model parameters were frozen.

Among the 770 training cases, 527 were sourced from the retrospective, multicentre PAIR-1 study conducted across six UK National Health Service hospitals using various scanner models and acquisition protocols; the full study protocol has been published previously [13]. The remaining 243 training cases were drawn from the PRIME study, a prospective, international, multicentre, within-patient diagnostic yield trial [15]. The inclusion of PRIME data aimed to enhance the model generalisability, and both studies mandated the inclusion of scans of high diagnostic quality. The prevalence of ≥ GG2 disease in the training set was 34%. In both datasets, Prostate Imaging-Reporting and Data Systems (PI-RADS) MRI acquisition and reporting guidelines [16] were followed for all included cases. Patient demographics, vendor characteristics, and image acquisition protocols are listed in Supplementary Information.

**Fig. 1:**
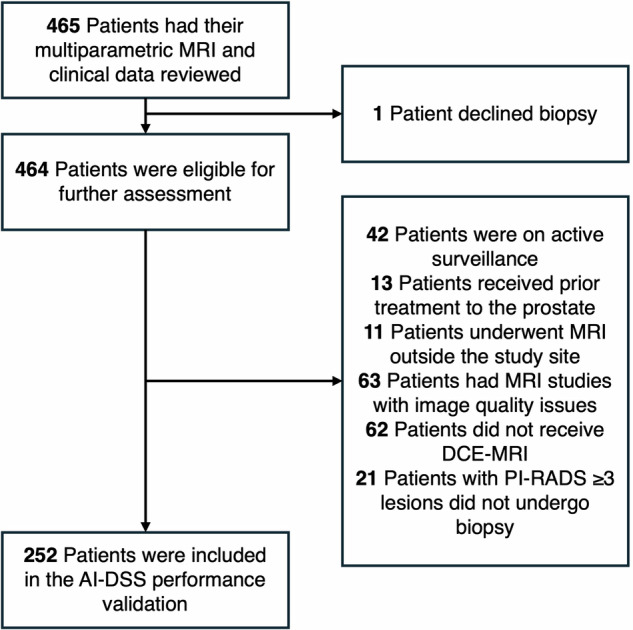
CONSORT diagram illustrating patient selection for the validation set. The validation set is similar to the one used in the original PAIR-1 study report [13]. AI-DSS, artificial intelligence-based decision support system; DCE-MRI, dynamic contrast-enhanced magnetic resonance imaging; PI-RADS, Prostate Imaging-Reporting and Data System

The 252 validation cases were from the PAIR-1 study [13], with the CONSORT diagram provided in Fig. 1. The prevalence of ≥ GG2 disease in this cohort was 31%.

### Ground truth

For all patients included in this study, a multidisciplinary team, including radiologists, determined the need for biopsy based on local standard-of-care procedures, with further details documented in PAIR-1 and PRIME study protocols [13, 17]. In cases suspicious of harbouring clinically significant PCa, both targeted and systematic biopsies were performed. Histopathological analysis of biopsy samples was used to confirm the presence of ≥ GG2 disease. Patients with negative MRI findings who did not undergo biopsy were assumed to be negative for clinically significant PCa.

### AI-DSS model development

AI-DSS was developed primarily to reduce false positives that lead to unproductive biopsies sampling either GG1 disease or benign tissue. The model outputs a continuous AI-DSS score indicating whether a patient should undergo a biopsy. This would typically be used with a threshold determined to minimise biopsies while maintaining the target ≥ GG2 cancer detection rate (CDR). To derive its prediction, the model integrates radiologists’ PI-RADS scores, automated prostate-specific antigen density (PSAd), and imaging-based cancer risk scores.

The imaging-based risk scores were generated using the proprietary Conformité Européenne (CE)-certified computer-aided detection medical device (Lucida Medical, Prostate Intelligence™-Pi-v3.0). Pi processes the axial images from either mpMRI or bpMRI scans to produce lesion- and patient-level risk scores on a continuous scale from 1 to 5. Pi is further described in [13] and in Supplementary Information.

The AI-DSS uses the following patient-level features in descending order of importance: PI-RADS score, MRI-AI risk score derived from the Pi v.3.0 model, and PSA density. These three features were used to train a machine learning-based risk calculator, with ≥ GG2 being the target label. Originally assigned PI-RADS scores were reviewed by two radiologists (at least one being at the Consultant level) prior to their incorporation into the classifier. PSAd was derived from automatic whole-volume prostate segmentations performed by Pi.

### AI-DSS model evaluation and benefit-to-harm ratio calculation

The trained AI-DSS model was applied to the hold-out validation cohort. Metrics used to assess the model performance included the CDR, number of ≥ GG2 cancers missed, number of GG1 cancers detected, grade selectivity, biopsy efficiency, and selective biopsy avoidance, with the appropriate formulae [12] presented below.

$${Grade}\,{selectivity}=\,\frac{\ge {GG}2\,{cancers}\,{detected}}{{GG}1\,{cancers}\,{detected}}$$

$${Biopsy}\,{efficiency}=\,\frac{\ge {GG}2\,{cancers}\,{detected}}{{GG}1\,{cancers}\,{detected}+{Benign}\,{biopsies}}$$

$${Selective}\,{biopsy}\,{avoidance}=\,\frac{\,{Avoided}\,{biopsies}}{{Benign}\,{biopsies}}$$

For comparison, the AI-DSS performance metrics were assessed against three scenarios. Scenario 1 follows the reference standard of care, i.e., the results of biopsy decisions made by the original multidisciplinary team that oversaw clinical care of the validation cohort. Scenario 2 mandated biopsy in all patients who had either PI-RADS ≥ 3 disease or PSAd ≥ 0.15. Scenario 3 mandated biopsy in patients who had either PI-RADS ≥ 4 disease or PSAd ≥ 0.15 in compliance with the NICE risk-based pathway [18]. In the latter two scenarios, PSAd values were derived from the original patient notes, while AI-DSS used PSAd derived using an AI-trained prostate segmentation algorithm. These scenarios were selected to benchmark the AI-DSS against commonly used clinical biopsy thresholds and assess its potential for reducing unnecessary biopsies at a pre-specified CDR.

### False-negative case assessment

For ≥ GG2 missed by AI-DSS, we retrieved the following clinical information from the electronic case report forms collected as part of the PAIR-1 study, in addition to PSAd, PI-RADS, and biopsy GG: radiological T-stage [19], percentage of Gleason pattern 4 disease (%GP4), maximum cancer core length (MCCL), presence of adverse pathology [20] (any Gleason pattern 5, large cribriform morphology or intraductal carcinoma, complex intraluminal papillary architecture, grade 3 stromogenic carcinoma, or complex anastomosing cord-like growth), along with the concordance between biopsy and MRI results. The rationale for this was to evaluate the potential aggressiveness of the false-negative cases and understand the limitations of the AI-DSS.

## Results

### Cohort characteristics

The mean age of patients in the validation cohort (*n* = 252) was 67.3 years (standard deviation: 8.5 years), and the median pre-biopsy PSA was 6.81 ng/mL (interquartile range: 4.73–10.62 ng/mL). Additional cohort characteristics have been detailed previously in the original PAIR-1 study report [13].

### Benchmark biopsy strategies and benefit-to-harm ratios

**Fig. 2:**
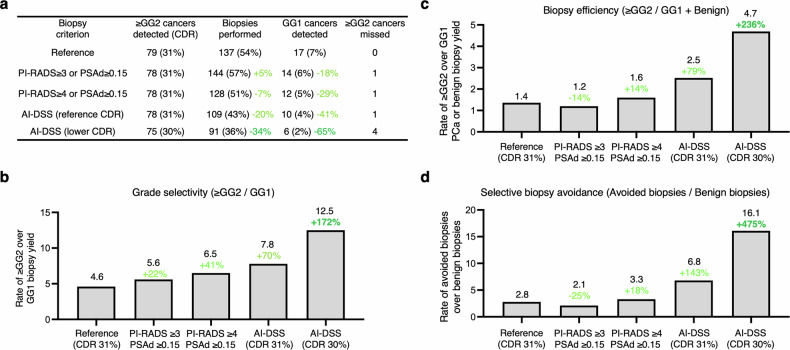
**a** Number and proportion of ≥ GG2 cancers detected, number of biopsies performed, number of GG1 cancers detected, and number of ≥ GG2 cancers missed by the reference standard, two clinical benchmark scenarios, and AI-DSS used at the reference (31%) and reduced (30%) CDR. **b**–**d** Box plots comparing grade selectivity (**b**), biopsy efficiency (**c**), and selective biopsy avoidance (**d**) ratios across the five biopsy recommendation strategies used in this study. AI-DSS, artificial intelligence-based decision support system; CDR, cancer detection rate; GG, grade group; PI-RADS, Prostate Imaging-Reporting and Data System; PSAd, prostate-specific antigen density

137/252 (54%) patients in the validation set underwent biopsy, resulting in the detection of ≥ GG2 and GG1 disease in 79/252 (31%) and 17/252 (7%) men, respectively (Fig. 2a). This reference standard yielded grade selectivity, biopsy efficiency, and selective biopsy avoidance of 4.6, 1.4, and 2.8, respectively (Fig. 2b–d).

If only patients with a PI-RADS ≥ 3 and/or PSAd ≥ 0.15 were biopsied while maintaining the CDR at 31%, this would result in seven additional biopsies, with three undetected GG1 tumours and one missed ≥ GG2 tumour (Fig. 2a). The resulting grade selectivity, biopsy efficiency, and selective biopsy avoidance would be 5.6 (+22%), 1.2 (−14%) and 2.1 (−25%), respectively (Fig. 2b–d).

If only patients with a PI-RADS ≥ 4 and/or PSAd ≥ 0.15 were biopsied at the CDR of 31%, this would result in nine fewer biopsies, five undetected GG1 tumours, and one missed ≥ GG2 tumour (Fig. 2a) compared to the reference standard. The resulting grade selectivity, biopsy efficiency, and selective biopsy avoidance would increase to 6.5 (+41%), 1.6 (+14%), and 3.3 (+18%), respectively (Fig. 2b–d).

### AI-DSS and benefit-to-harm ratios

Compared to the reference standard, using AI-DSS at a 31% CDR would result in 28 fewer biopsies, seven undetected GG1 tumours, and one missed ≥ GG2 tumour (Fig. 2a). The same ≥ GG2 would be missed also by scenarios 2 and 3. The resulting grade selectivity, biopsy efficiency, and selective biopsy avoidance would increase to 7.8 (+66%), 2.5 (+79%), and 6.8 (+143%), respectively (Fig. 2b–d). Of the 28 patients whose biopsies could have been avoided, 13 (46%) and 15 (54%) had PI-RADS 2 and PI-RADS 3 lesions, respectively.

Notably, using AI-DSS at a slightly reduced 30% CDR would result in marked improvements in the benefit-to-harm ratios than the reference standard, with 46 fewer biopsies, 11 undetected GG1 tumours; however, missing four ≥ GG2 tumours compared to the reference standard (Fig. 2a). The resulting grade selectivity, biopsy efficiency, and selective biopsy avoidance would increase to 12.5 (+172%), 4.7 (+236%), and 16.1 (+475%), respectively (Fig. 2b–d). Of the 46 patients whose biopsies could have been avoided under this strategy, 13 (28%), 26 (56%), and 7 (16%) had PI-RADS 2, PI-RADS 3, and PI-RADS 4 lesions, respectively.

### False-negative and true-negative AI-DSS cases

**Fig. 3:**
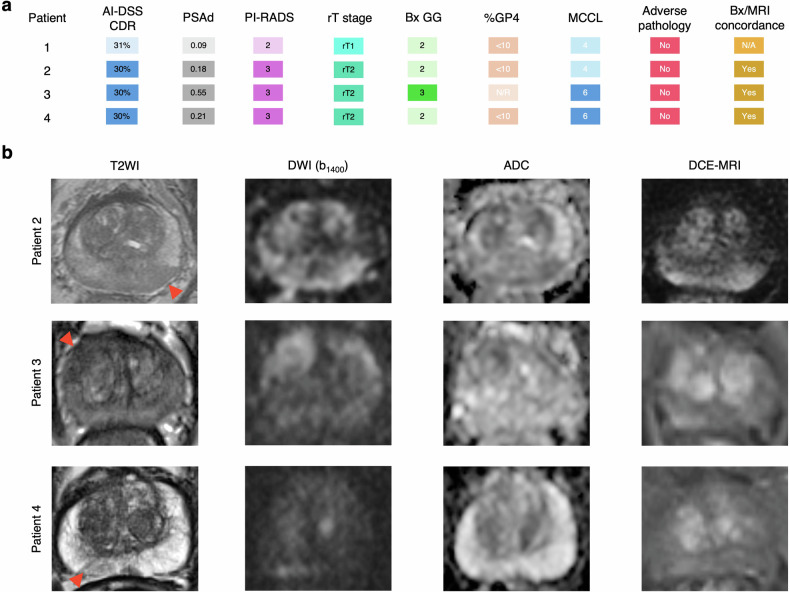
**a** Summary characteristics of ≥ GG2 missed by AI-DSS at the reference (31%) and reduced (30%) CDR. **b** Representative multiparametric MR images of the three patients with originally reported ≥ GG2 PI-RADS 3 lesions (red arrows), which would have been missed by AI-DSS at 30% CDR. AI-DSS, artificial intelligence-based decision support system; Bx, biopsy; CDR, cancer detection rate; GG, grade group; %GP4, percentage of Gleason pattern 4 disease; MCCL, maximum cancer core length in mm; PI-RADS, Prostate Imaging-Reporting and Data System; PSAd, prostate-specific antigen density; ADC, apparent diffusion coefficient; DCE-MRI, dynamic contrast-enhanced magnetic resonance imaging; DWI, diffusion-weighted imaging

**Fig. 4:**
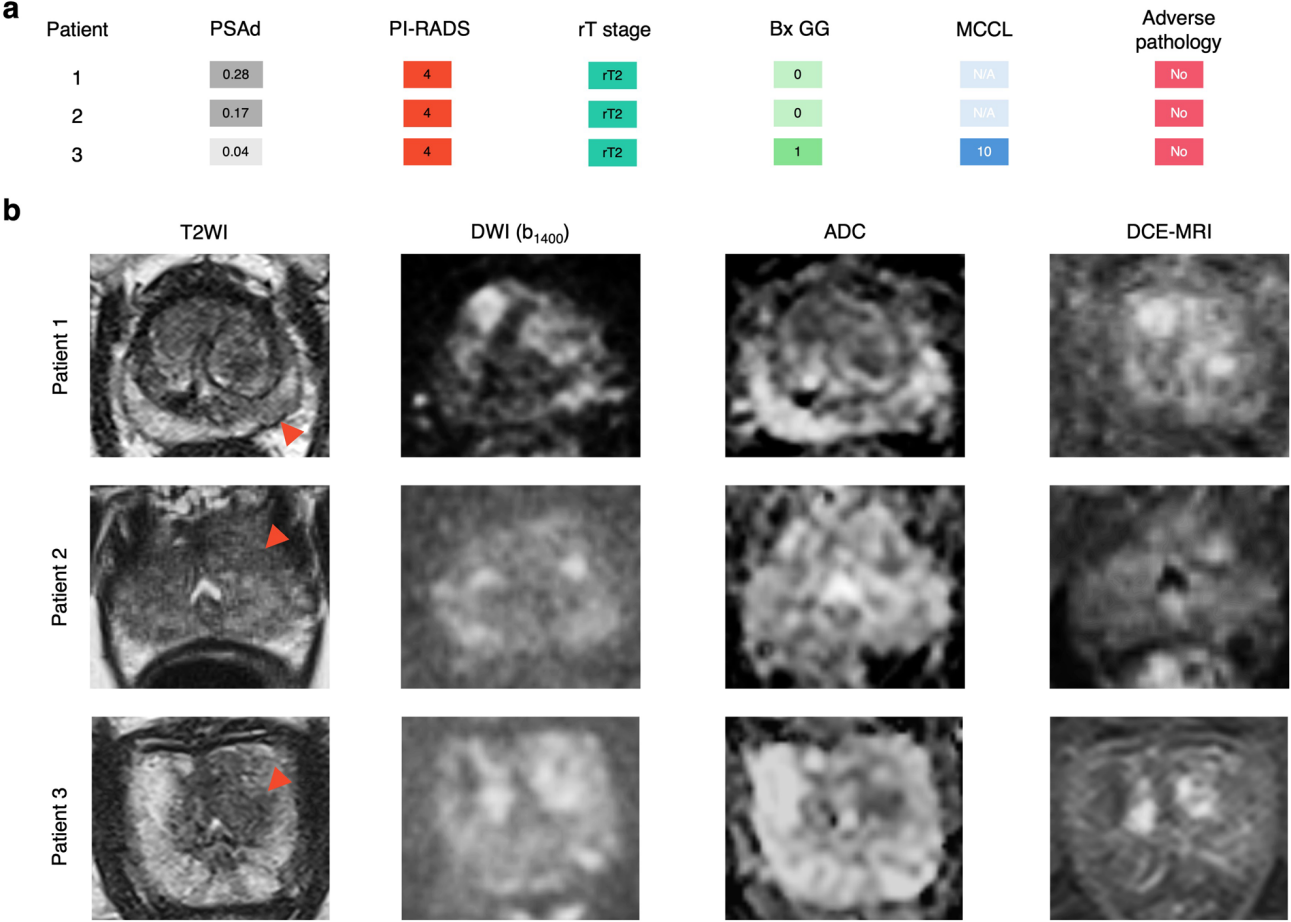
**a** Summary characteristics of PI-RADS 4 cases with benign or GG1-yielding biopsies that were classified as true negative by AI-DSS at 30% CDR. **b** Representative multiparametric MR images of the three patients with originally reported PI-RADS 4 lesions (red arrows), which would not have been biopsied if AI-DSS classification was followed. AI-DSS, artificial intelligence-based decision support system; Bx, biopsy; GG, grade group; MCCL, maximum cancer core length in mm; PI-RADS, Prostate Imaging-Reporting and Data System; PSAd, prostate-specific antigen density; ADC, apparent diffusion coefficient; DCE-MRI, dynamic contrast-enhanced magnetic resonance imaging; DWI, diffusion-weighted imaging

Clinical and radiological characteristics of the four ≥ GG2 tumours missed by AI-DSS at 30% CDR are presented in Fig. 3. Besides Patient 1, who did not have MRI-visible disease reported clinically (PI-RADS 2) and had low PSAd of 0.09, the other three patients had either equivocal MRI-visible lesions (originally reported as PI-RADS 3) or PSAd ≥ 0.15 (Fig. 3a). In patients with MRI-visible lesions, biopsy showed tumour foci in areas concordant with the MRI reports (Fig. 3b). Notably, the maximum cancer core length in these foci did not exceed 6 mm, and none of the lesions were recorded to harbour adverse histological phenotypes. In addition, Fig. 4 presents examples of AI-DSS true negative cases that were originally reported as PI-RADS 4 and underwent biopsies yielding benign findings or GG1 disease.

## Discussion

In this multicentre, multiscanner study, we developed and retrospectively validated an AI-DSS designed to optimise prostate biopsy decision-making. Our primary finding is that the AI-DSS, which integrates PI-RADS scores, automated PSAd calculations, and deep-learning-derived imaging risk scores, may substantially improve biopsy benefit-to-harm ratios compared to current standard-of-care and other common biopsy decision thresholds. Specifically, when benchmarked against the real-world decisions made in our validation cohort, the AI-DSS could have avoided 28 biopsies while missing only one additional ≥ GG2 cancer, thereby increasing biopsy efficiency by 79% and grade selectivity by 70%. A 1% reduction in ≥ GG2 CDR compared to the reference standard would have enabled AI-DSS to avoid 46 biopsies, substantially increasing biopsy efficiency by 236% and grade selectivity by 172%, missing a total of four ≥ GG2 cancers. Notably, the latter approach offers a considerable improvement in all biopsy benefit-to-harm ratios compared to the NICE risk-based pathway [18] threshold of PI-RADS ≥ 4 and/or PSAd ≥ 0.15.

If the current MRI-based PCa diagnostic pathway remains the standard of care and unchanged, the doubling of PCa incidence by 2040 [6] will overwhelm radiology departments, which already struggle with severe workforce shortages. As the potential implementation of MRI-based PCa population screening is gaining traction, the growing number of patients recalled for full MRI examination risks adding further pressure on the early cancer diagnosis system. Critically, as the diagnostic pathway is increasingly focusing on maximising the detection of ≥ GG2 disease, efficient biopsy decision-making is imperative for the assessment of its success.

Our findings demonstrate the potential of AI-DSS to deliver tangible improvements in biopsy decision-making by identifying more patients unlikely to harbour benign or GG1 pathology. Avoiding biopsies in these men could substantially improve the pathway in terms of grade selectivity, biopsy efficiency, and selective biopsy avoidance, metrics designed to evaluate the efficiency of MRI-based PCa screening strategies [7]. It is important to note that safely avoiding immediate biopsies, particularly for MRI-visible (PI-RADS ≥ 3) lesions, will necessitate robust follow-up protocols, which will likely increase the demand for surveillance MRIs. This scenario reinforces the critical need for tools that improve pathway efficiency, as overall imaging demand is set to rise from new referrals, active surveillance protocols, and follow-up scanning for those who avoid biopsy.

Practically, integrating an AI-DSS like ours into the clinical workflow as an automated “second reader” could provide the multidisciplinary team with an independent, quantitative risk score to supplement the radiologist’s report and clinical data. This could help standardise biopsy recommendations across institutions, mitigating the impact of reader experience and potentially improving equity of access to expert-level interpretation. Furthermore, by automating prostate volume and PSAd calculation, the AI-DSS could reduce manual workload and improve consistency. In a screening scenario, the AI-DSS could function as a triage tool, ranking cases based on their probability of harbouring ≥ GG2 disease.

Importantly, setting up a CDR that is appropriate to the clinical setting and population characteristics enables further refinement of the AI-DSS performance. In this study, using AI-DSS at the reference CDR led to missing one MRI-invisible (PI-RADS 2), 4 mm GG2 cancer without any adverse histology in the biopsy specimen and with low reported PSAd (0.09). The dramatic increase in biopsy efficiency achieved by reducing the CDR by 1% came at the expense of missing three additional ≥ GG2 cancers by AI-DSS. All three had indeterminate findings on mpMRI (PI-RADS 3) and ≤ 6 mm tumours on biopsy (with small volume potentially hindering the reliability of tumour grading), again without any adverse histology. Considering the excellent 15-year disease-specific survival of clinically localised disease in the ProtecT trial [21], favourable outcomes of non-cribriform GG2 disease in ProtecT [22] and on contemporary active surveillance [23], and the lack of adverse histology in these cases, there is increasing evidence that these patients would not experience adverse outcomes in the intermediate term.

However, these results highlight that any strategy using AI-DSS to defer biopsies in men with MRI-visible disease must be coupled with a robust surveillance protocol. Developing such a protocol is challenging, considering the lack of universally accepted MRI-driven active surveillance programmes even for biopsy-proven disease. However, one prospective approach, which has proven safe for monitoring men on active surveillance, could be to offer quarterly PSA testing for a defined period (e.g., 3 years) after the omission of biopsy, supplemented by a low threshold for repeat MRI if clinically indicated [24]. Crucially, the aforementioned ≥ GG2 cases would have been missed if AI-DSS were used as a standalone biopsy decision-making tool. However, its intended use is as part of the real-world clinical workflow (radiological decision support). Using AI-DSS in conjunction with a comprehensive assessment of clinical factors by the multidisciplinary team is likely to miss fewer ≥ GG2 cases while maintaining reductions in false positives. Testing this prospectively as part of the clinical workflow is the key objective for future studies.

Here, the retrospective nature of the study is among its key limitations. Verification bias is also a factor, as the ground truth for ≥ GG2 cancers was primarily available for patients who underwent biopsy based on existing protocols. Patients with negative MRI findings who did not undergo biopsy were assumed to be negative for ≥ GG2 disease, which is a common limitation for AI-validation studies in the modern era. Hence, the focus of this evaluation was to perform a comparative analysis against the standard of care (no biopsy for negative cases unless clinically indicated) by focusing on the clinical impacts of the MRI pathways. Importantly, future work will aim to improve the generalisability of the developed AI-DSS by expanding the development and validation datasets through a larger cohort size and wider representation of different vendors.

Given these limitations, robust prospective validation is essential before clinical implementation. One scenario would be a prospective cohort study where AI-DSS is run in the background on all patients undergoing pre-biopsy MRI. Clinical teams would remain blinded to the AI recommendations, allowing for a direct, real-world comparison of the AI-DSS’s performance against actual clinical outcomes without affecting patient care. The second, and more definitive, approach would be a prospective randomised controlled trial, or at least a within-patient study similar to PRIME [17]. In such a trial, patients would be randomised to either a standard-of-care arm (where the MDT makes decisions without AI input) or an AI-assisted arm (where the MDT is provided with the AI-DSS report). The primary endpoints would be on comparative pathway outputs: biopsy efficiency, grade selectivity, and selective biopsy avoidance, with an active and programmatic follow-up of men who avoided biopsy in the AI-assisted arm to monitor for disease misclassification. Such a trial would provide the highest level of evidence to confirm whether this AI-DSS can safely and effectively improve upon the current prostate cancer diagnostic pathway.

In conclusion, our study demonstrates that an AI-DSS integrating clinical and advanced imaging data can improve the benefit-to-harm ratio of prostate biopsy decisions in a retrospective setting. By enhancing grade selectivity and biopsy efficiency, this technology holds promise for optimising the diagnostic pathway, particularly in the face of rising demand and the potential advent of population screening. However, its clinical utility and safety must be confirmed in prospective trials before it can be recommended for clinical adoption.

## Supplementary information

ELECTRONIC SUPPLEMENTARY MATERIAL

## Abbreviations

AI-DSS: Artificial intelligence-based decision support system
GG: Grade group
MCCL: Maximum cancer core length
PCa: Prostate cancer
PSAd: Prostate-specific antigen density

## References

[1] Cornford P, van den Bergh RCN, Briers E et al (2024) EAU-EANM-ESTRO-ESUR-ISUP-SIOG guidelines on prostate cancer—2024 update. Part I: Screening, diagnosis, and local treatment with curative intent. Eur Urol 86:148–163 38614820 10.1016/j.eururo.2024.03.027

[2] Wei JT, Barocas D, Carlsson S et al (2023) Early detection of prostate cancer: AUA/SUO guideline Part II: Considerations for a prostate biopsy. J Urol 210:54–63 37096575 10.1097/JU.0000000000003492 PMC11321723

[3] Mason RJ, Marzouk K, Finelli A et al (2022) UPDATE—2022 Canadian Urological Association recommendations on prostate cancer screening and early diagnosis: endorsement of the 2021 Cancer Care Ontario guidelines on prostate multiparametric magnetic resonance imaging. Can Urol Assoc J 16:E184–E196 35358414 10.5489/cuaj.7851 PMC9054332

[4] Soerensen SJC, Li S, Langston ME, Fan RE, Rusu M, Sonn GA (2024) Trends in pre-biopsy MRI usage for prostate cancer detection, 2007–2022. Prostate Cancer Prostatic Dis 28:519–522 39306635 10.1038/s41391-024-00896-y PMC11925800

[5] Sushentsev N, Comune R, Sinci KA et al (2025) A 10-year analysis of MRI-driven prostate cancer diagnosis and active surveillance: trends and implications. BJU Int. 10.1111/BJU.16743 10.1111/bju.16743 PMC12256747 40223647

[6] James ND, Tannock I, N’Dow J et al (2024) The Lancet Commission on prostate cancer: planning for the surge in cases. Lancet 403:1683–1722 38583453 10.1016/S0140-6736(24)00651-2 PMC7617369

[7] Schoots IG, Padhani AR (2025) A paradigm shift toward population-based MRI-targeted prostate cancer screening. JAMA Oncol. 10.1001/JAMAONCOL.2025.1594 10.1001/jamaoncol.2025.1594 40531480

[8] Christensen EW, Parikh JR, Drake AR, Rubin EM, Rula EY (2025) Projected US radiologist supply, 2025 to 2055. J Am Coll Radiol 22:161–169 39952776 10.1016/j.jacr.2024.10.019

[9] Barrett T, Ghafoor S, Gupta RT et al (2022) Prostate MRI qualification: *AJR* Expert Panel narrative review. AJR Am J Roentgenol 219:691–702 35544372 10.2214/AJR.22.27615

[10] Padhani AR, Godtman RA, Schoots IG (2024) Key learning on the promise and limitations of MRI in prostate cancer screening. Eur Radiol 34:6168–6174 38311703 10.1007/s00330-024-10626-6

[11] Westphalen AC, McCulloch CE, Anaokar JM et al (2020) Variability of the positive predictive value of PI-RADS for prostate MRI across 26 centers: experience of the Society of Abdominal Radiology prostate cancer disease-focused panel. Radiology 296:76–84 32315265 10.1148/radiol.2020190646 PMC7373346

[12] Schoots IG, Ahmed HU, Albers P et al (2025) Magnetic resonance imaging–based biopsy strategies in prostate cancer screening: a systematic review. Eur Urol. 10.1016/J.EURURO.2025.05.038 10.1016/j.eururo.2025.05.038 40514255

[13] Giganti F, Moreira da Silva N, Yeung M et al (2025) AI-powered prostate cancer detection: a multi-centre, multi-scanner validation study. Eur Radiol 35:4915–4924 10.1007/s00330-024-11323-0 PMC12226644 40016318

[14] Saha A, Bosma JS, Twilt JJ et al (2024) Artificial intelligence and radiologists in prostate cancer detection on MRI (PI-CAI): an international, paired, non-inferiority, confirmatory study. Lancet Oncol 25:879–887 38876123 10.1016/S1470-2045(24)00220-1 PMC11587881

[15] Asif A, Nathan A, Ng A et al (2023) Comparing biparametric to multiparametric MRI in the diagnosis of clinically significant prostate cancer in biopsy-naive men (PRIME): a prospective, international, multicentre, non-inferiority within-patient, diagnostic yield trial protocol. BMJ Open. 10.1136/BMJOPEN-2022-070280 10.1136/bmjopen-2022-070280 PMC10083803 37019486

[16] Turkbey B, Rosenkrantz AB, Haider MA et al (2019) Prostate Imaging Reporting and Data System version 2.1: 2019 update of Prostate Imaging Reporting and Data System Version 2. Eur Urol 76:340–351 30898406 10.1016/j.eururo.2019.02.033

[17] Ng AB, Asif A, Agarwal R et al (2025) Biparametric vs Multiparametric MRI for Prostate Cancer Diagnosis: The PRIME Diagnostic Clinical Trial. JAMA. 334:1170–1179 10.1001/jama.2025.13722 PMC12423955 40928788

[18] NICE (2019) Prostate cancer: diagnosis and management. Available via https://www.nice.org.uk/guidance/ng131/chapter/Recommendations. Accessed 4 Aug 2025

[19] Sushentsev N, Barrett T, Warren AY, Gnanapragasam VJ (2022) How and when should radiologists report T-staging on MRI in patients with prostate cancer? BJU Int. 10.1111/BJU.15824 10.1111/bju.15824 35696280

[20] Nguyen JK, Harik LR, Klein EA et al (2024) Proposal for an optimised definition of adverse pathology (unfavourable histology) that predicts metastatic risk in prostatic adenocarcinoma independent of grade group and pathological stage. Histopathology 85:598–613 38828674 10.1111/his.15231 PMC11365761

[21] Hamdy FC, Donovan JL, Lane JA et al (2023) Fifteen-year outcomes after monitoring, surgery, or radiotherapy for prostate cancer. N Engl J Med 388:1547–1558 36912538 10.1056/NEJMoa2214122

[22] Sushentsev N, Warren AY, Colling R et al (2025) Active Monitoring, Surgery, and Radiotherapy for Cribriform-Positive and Cribriform-Negative Prostate Cancer: A Secondary Analysis of the PROTECT Randomized Clinical Trial. JAMA Oncol. 11:1512–1517 10.1001/jamaoncol.2025.4125 PMC12532030 41100113

[23] Sushentsev N, Li IG, Xu G et al (2025) Predicting active surveillance failure for patients with prostate cancer in the magnetic resonance imaging era: a multicentre transatlantic cohort study. Eur Urol Oncol. 10.1016/J.EUO.2025.06.012 10.1016/j.euo.2025.06.012 40645823

[24] Hamm CA, Asbach P, Pöhlmann A et al (2025) Oncological safety of MRI-informed biopsy decision-making in men with suspected prostate cancer. JAMA Oncol 11:145–153 39666360 10.1001/jamaoncol.2024.5497 PMC11843366

