# Supplementary material for: AI decision support for increasing prostate biopsy efficiency: a retrospective multicentre, multiscanner study
Source: Eur Radiol. 2026 Feb 20;36(7):5719–27. doi: 10.1007/s00330-026-12361-6 (PMC13282241; doi:10.1007/s00330-026-12361-6)
Supplement: Supplementary file 1 — ELECTRONIC SUPPLEMENTARY MATERIAL [file 330_2026_12361_MOESM1_ESM.pdf]

AI decision support for increasing prostate biopsy efficiency: a retrospective multicentre, multi-scanner study

ELECTRONIC SUPPLEMENTARY MATERIAL

Supplementary Methods

In this study, a total of 815 cases (with a 35% prevalence of csPCa) were used for development of the Pi v3.0 MRI-AI model. The development dataset consisted of 612 cases from five NHS hospitals in the PAIR-1 study and 203 cases from the public dataset PROSTATEx. The MRI-AI model and its development have been described previously [1]. In [1], the performance of the model for clinically significant prostate cancer detection was found to be statistically non-inferior to radiologists, with a 10% performance margin considered acceptable. The model attained an AUC of 0.91, compared to 0.95 for radiologists. At the predefined risk threshold of 3.5, the model achieved a sensitivity of 95% and a specificity of 67%. In comparison, radiologists using PI-RADS/Likert ≥3 identified Grade Group ≥2 cancers with a sensitivity of 99% and specificity of 73%.

Supplementary references:

1. Giganti F, Moreira da Silva N, Yeung M, et al (2025) AI-powered prostate cancer detection: a multi-centre, multi-scanner validation study. Eur Radiol 1–10. https://doi.org/10.1007/S00330-024-11323-0/FIGURES/3

Supplementary Table 1 - Number of patients and MR data for each centre.
Legend – MR: Magnetic Resonance; GEHC: GE HealthCare; T: Tesla. All cases used a PI-RADS compliant multiparametric MR protocol comprising at least axial T2 weighted imaging, diffusion-weighted imaging, and dynamic contrast enhanced imaging.

	Number of patients (Validation set)	Number of patients (AI-DSS Development set)	Prevalence of GG>2 (Validation set)	MR scanner vendor	Field strength
PAIR-1 Site 1	42	90	45%	Siemens	1.5
PAIR-1 Site 2	42	111	24%	Siemens	3T
PAIR-1 Site 3	42	86	26%	Siemens	3T
PAIR-1 Site 4	42	0	33%	Siemens	1.5T
PAIR-1 Site 5	42	128	33%	GEHC	3T
PAIR-1 Site 6	42	112	26%	Siemens	1.5T (n=30)<br>3T (n=12)
PRIME Site A	N/A	14	N/A	Siemens	3T
PRIME Site B	N/A	28	N/A	Siemens	3T
PRIME Site C	N/A	22	N/A	Siemens	3T
PRIME Site D	N/A	5	N/A	Siemens	3T
PRIME Site E	N/A	26	N/A	GEHC	3T
PRIME Site F	N/A	9	N/A	Siemens	3T
PRIME Site G	N/A	11	N/A	Siemens	3T
PRIME Site H	N/A	8	N/A	Philips	3T
PRIME Site I	N/A	13	N/A	Siemens	1.5T
PRIME Site J	N/A	8	N/A	Siemens	3T
PRIME Site K	N/A	40	N/A	Siemens (n=7)<br>Philips (n=33)	1.5T (n=7)<br>3T (n=33)
PRIME Site L	N/A	25	N/A	Siemens	3T
PRIME Site M	N/A	29	N/A	Siemens	3T
PRIME Site N	N/A	1	N/A	Siemens	3T
PRIME Site O	N/A	4	N/A	Philips	3T

Supplementary Table 2 – Demographic characteristics for the validation set (N = 252).

Validation set		csPCa	non-csPCa
Total (N)		79	173
Age mean ± std		69.4±7.8	65.8±8.6
PSA (ng/mL) median (IQR)		10.2 (IQR: 6.3 - 14.5)	5.9 (IQR: 4.1-8.8)
BPH (N)		1*1	16*2
Prostatitis (N)		0*3	3*4
UTI (N)		2*5	10*6
Family history<br>Genetic predisposition (N)		11*7	24*8
LUTS (N)		44*9	91*10
Prostate Volume (mL) mean ± std		50.2±26.9	71.5±39.0
PI-RADS (N)	1	0	4
	2	1	123
	3	4	23
	4	22	17
	5	52	6
Gleason Grade Group (N)	Benign	0	41
	No biopsy	0	115
	1	0	17
	2	41	0
	3	18	0
	4	5	0
	5	15	0

Missing data: *1 - 26 patients without BPH information, *2 - 57 patients without BPH information, *3 - 26 patients without Prostatitis information, *4 - 62 patients without Prostatitis information, *5 - 26 patients without UTI information, *6 - 62 patients without UTI information, *7 - 21 patients without FH/GP information, *8 - 55 patients without FH/GP information, *9 - 15 patients without LUTS information, *10 - 50 patients without LUTS information

Supplementary Table 3 – Demographic characteristics per site for the validation set (N = 252).

Demographics		Validation					
		Site 1	Site 2	Site 3	Site 4	Site 5	Site 6
**Age mean ± std**		70.6±7.1	66.2±9.3	63.0±5.9	70.3±7.3	65.8±9.7	65.8±9.2
**Prostate Volume (mL) mean ± std**		72.0±36.7	59.1±40.6	59.2±20.6	73.9±42.3	65.4±38.4	59.3±38.5
**PSA level (ng/mL) median (IQR**		9.0 (IQR: 5.7 – 13.5)	6.0 (IQR: 2.9 – 8.4)	5.5 (IQR: 4.1 – 7.1)	9.4 (IQR: 6.4 – 15.1)	6.3 (IQR: 4.6 – 9.4)	6.6 (IQR: 4.4 – 10.0)
**PI-RADS (N)**	1	0	0	0	0	0	4
	2	21	22	21	21	20	19
	3	2	5	11	1	3	5
	4	4	3	6	11	7	8
	5	15	12	4	9	12	6
**Gleason Grade Group (N)**	Benign	1	7	13	4	9	7
	No biopsy	21	18	17	19	18	22
	1	1	7	1	5	1	2
	2	8	2	7	8	9	7
	3	6	3	2	3	3	1
	4	1	0	2	1	0	1
	5	4	5	0	2	2	2

Supplementary references:

1. Giganti F, Moreira da Silva N, Yeung M, et al (2025) AI-powered prostate cancer detection: a multi-centre, multi-scanner validation study. Eur Radiol 1–10. https://doi.org/10.1007/S00330-024-11323-0/FIGURES/3
